# Deep Learning for Automated Contouring of Gross Tumor Volumes in Esophageal Cancer

**DOI:** 10.3389/fonc.2022.892171

**Published:** 2022-07-18

**Authors:** Linzhi Jin, Qi Chen, Aiwei Shi, Xiaomin Wang, Runchuan Ren, Anping Zheng, Ping Song, Yaowen Zhang, Nan Wang, Chenyu Wang, Nengchao Wang, Xinyu Cheng, Shaobin Wang, Hong Ge

**Affiliations:** ^1^ Department of Radiation Oncology, The Affiliated Cancer Hospital of Zhengzhou University & Henan Cancer Hospital, Zhengzhou, China; ^2^ Department of Radiation Oncology, Anyang Tumor Hospital, The Fourth Affiliated Hospital of Henan University of Science and Technology, Anyang, China; ^3^ Department of Research and Development, MedMind Technology Co, Ltd., Beijing, China

**Keywords:** deep learning, automated contouring, esophageal cancer, gross tumor volumes, radiotherapy

## Abstract

**Purpose:**

The aim of this study was to propose and evaluate a novel three-dimensional (3D) V-Net and two-dimensional (2D) U-Net mixed (VUMix-Net) architecture for a fully automatic and accurate gross tumor volume (GTV) in esophageal cancer (EC)–delineated contours.

**Methods:**

We collected the computed tomography (CT) scans of 215 EC patients. 3D V-Net, 2D U-Net, and VUMix-Net were developed and further applied simultaneously to delineate GTVs. The Dice similarity coefficient (DSC) and 95th-percentile Hausdorff distance (95HD) were used as quantitative metrics to evaluate the performance of the three models in ECs from different segments. The CT data of 20 patients were randomly selected as the ground truth (GT) masks, and the corresponding delineation results were generated by artificial intelligence (AI). Score differences between the two groups (GT versus AI) and the evaluation consistency were compared.

**Results:**

In all patients, there was a significant difference in the 2D DSCs from U-Net, V-Net, and VUMix-Net (p=0.01). In addition, VUMix-Net showed achieved better 3D-DSC and 95HD values. There was a significant difference among the 3D-DSC (mean ± STD) and 95HD values for upper-, middle-, and lower-segment EC (p<0.001), and the middle EC values were the best. In middle-segment EC, VUMix-Net achieved the highest 2D-DSC values (p<0.001) and lowest 95HD values (p=0.044).

**Conclusion:**

The new model (VUMix-Net) showed certain advantages in delineating the GTVs of EC. Additionally, it can generate the GTVs of EC that meet clinical requirements and have the same quality as human-generated contours. The system demonstrated the best performance for the ECs of the middle segment.

## Introduction

More than one-third of patients who have unresectable esophageal cancer (EC) or are ineligible for surgery are recommended for radiotherapy (RT) as the locoregional treatment modality (1). Accurate segmentation of the gross tumor volume (GTV) is critical for increasing the treatment efficacy. GTV contouring for EC is variable because the esophagus lacks a serous membrane; thus, there are differences in the quality, efficiency, and repeatability of delineation between different physicians (2). Additionally, the time to delineate a patient’s target area is affected by the physician’s proficiency, ranging from tens of minutes to 1 h. The automation of tumor contouring is an effective means to solve the above problems, aiming to improve the consistency and save time. However, the automation of tumor contouring for EC is challenging due to the substantial interpatient heterogeneity in tumor shape and the poorly defined tumor-to-normal tissue interface.

Artificial intelligence (AI) techniques using convolutional neural networks (CNNs) have shown tremendous potential in medical image processing and the automated segmentation of normal anatomy (3–8) and the GTV (9–12). These technologies have high accuracy and reduced processing time compared to the atlas-based segmentation methods (13, 14) commonly used today. As a well-known CNN architecture for medical image segmentation, U-Net (15) has been widely utilized in all CNN-based contour delineation models.

Recently, deep learning has achieved success in the automatic contouring of head and neck carcinoma (16–20), rectal carcinoma (21–23), breast carcinoma (24, 25), and cervical carcinoma tumors (26, 27). Nevertheless, there is little research on the application of CNNs in the delineation of the GTV for patients undergoing their initial EC treatment. Here, we present a novel three-dimensional (3D) V-Net (28) and two-dimensional (2D) U-Net mixed (VUMix-Net) architecture for segmentation of the GTV in the planning computed tomography (CT) for EC. We took advantage of two deep learning models to automatically delineate the GTV contour of EC. The first model yielded the gross localization of the EC, which was used to determine the slices containing the EC tumors. For each of these slices, the second model delineated the GTV contour precisely. We compared the performance for EC in the three segments of the esophagus and explored in which segment the application effect of the AI was best.

## Materials and methods

### Data and Preprocessing

The study was approved by the ethics committee of Anyang Cancer Hospital & The Fourth Affiliated Hospital and College of Clinical Medicine of Henan University Science and Technology. The patients provided written informed consent to participate in this study. The CT data from 215 patients with locally advanced EC were collected from November 2017 to January 2020. All scans were acquired with a Brilliance CT Big Bore (Philips Healthcare, Best, the Netherlands) with a thickness of 5 mm. The private information of the patients was kept confidential during the data collection and processing. The GTV contours delineated manually by a trained radiation oncologist (with more than 20 years of experience in caring for patients with EC) before radiotherapy in clinical practice were used as the segmentation ground truths (GTs). The GTV was defined according to CT imaging, gastroscopy, and esophagography findings. GTV delineation is associated with the International Commission on Radiology Radiation Units and Measurements 95 report (29) requirements, which are consistent, and delineation with reference to Chinese guidelines for the radiotherapy of esophageal cancer (2021 Edition) (30). To ensure the delineation quality, all the delineated contours were reviewed and modified (only when needed) together by a professional radiation oncologist committee consisting of six oncologists with more than 10 years of experience in radiotherapy for chest tumors, which is the clinical routine in the radiotherapy department of this hospital. These patients were randomly assigned to two cohorts: (a) a training–validation cohort of 185 patients and (b) a testing cohort of 30 patients.

### Network Architectures

Physicians have difficulty delineating the GTV because the location and shape of the EC tumor are highly variable and the boundaries are rarely clear.

**Figure 1:**
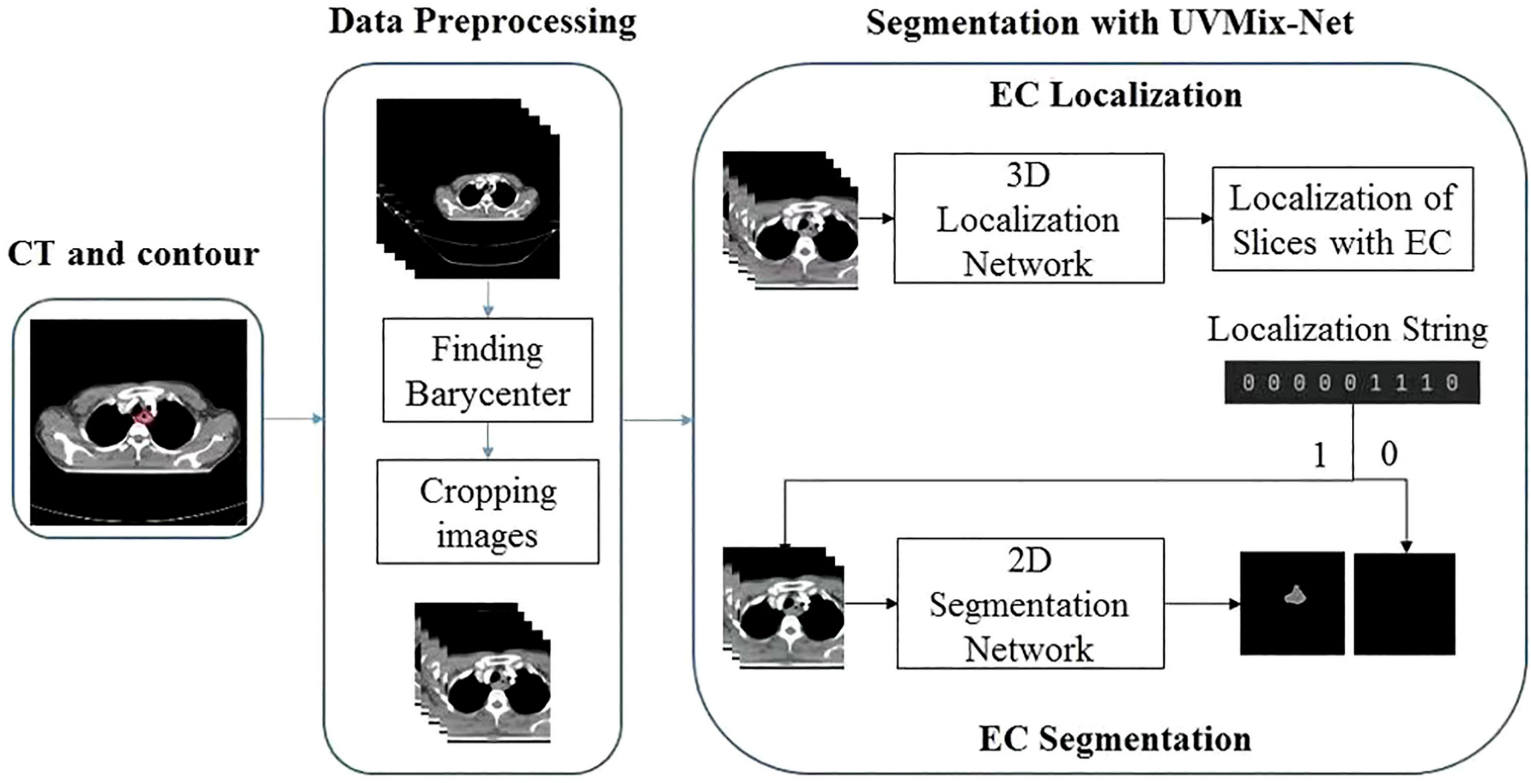
Architecture of VUMix-Net. The input CT slices are first cropped to better localize the body region. Slices are classified into positive or negative samples by the 3D V-Net-based CNN. Precise segmentation is further applied to the positive slices through the 2D U-Net-based CNN.

**Figure 2:**
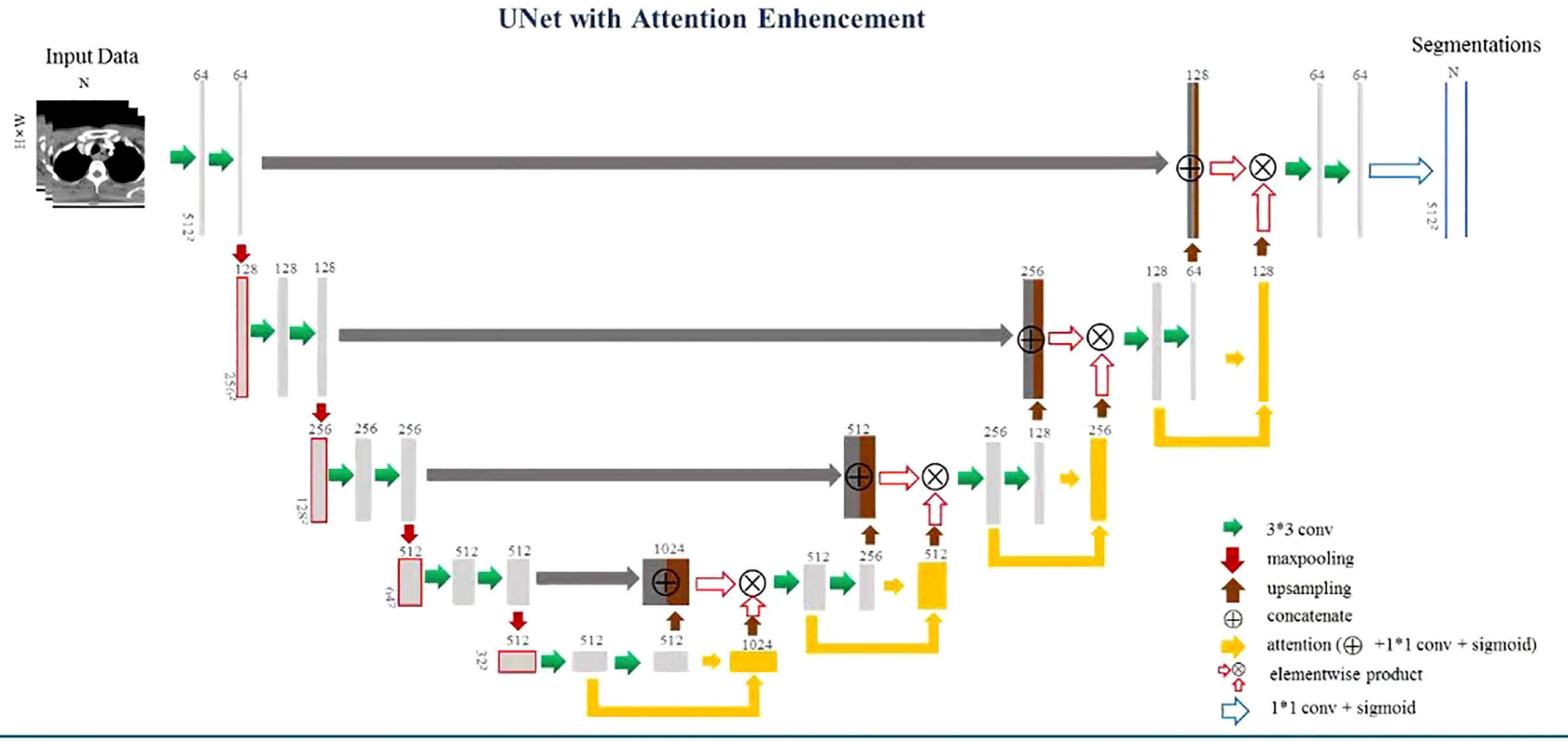
Architecture of the 2D U-Net-based CNN.

Therefore, to precisely identify the CT slices containing the EC tumor, we proposed a multiscale focusing strategy to segment the EC mask from coarse to fine. **Figure 1** shows the architecture of VUMix-Net. First, we converted all the CT scans to the same resolution of 1 mm × 1 mm × 5 mm. Second, each slice was cropped to a size of 192 × 192 with the barycenter of the body region as the center point. V-Net is implemented as a localization network to localize the EC tumor by extracting 3D-structured information with 3D convolutions with volumetric kernels and using residual blocks to avoid the vanishing gradient problem. The output of this 3D V-Net is then processed by analyzing the continuity of the positive slices. Only the slices within ten consecutive positive samples are input into the 2D segmentation network. **Figure 2** shows the architecture of the 2D U-Net-based CNN. A 2D U-Net backbone architecture consisting of an encoding path and a decoding path is used in the 2D segmentation network. To concatenate the multilevel features and take advantage of both the low-level and high-level information, the encoding path and decoding path are combined by a skip connection in the typical U-Net. As a result, the network learns to use the features equally. In this network, an attention block is proposed to weight the lower-level features to multiply with the higher-level features in the decoder part. Through the attention block, multilevel features are combined with skip connections. As a result, each attention block infers attention maps along both the channel dimension and spatial dimension for adaptive feature refinement. Informative features are emphasized, while other features are suppressed.

### Training

**Figure 3:**
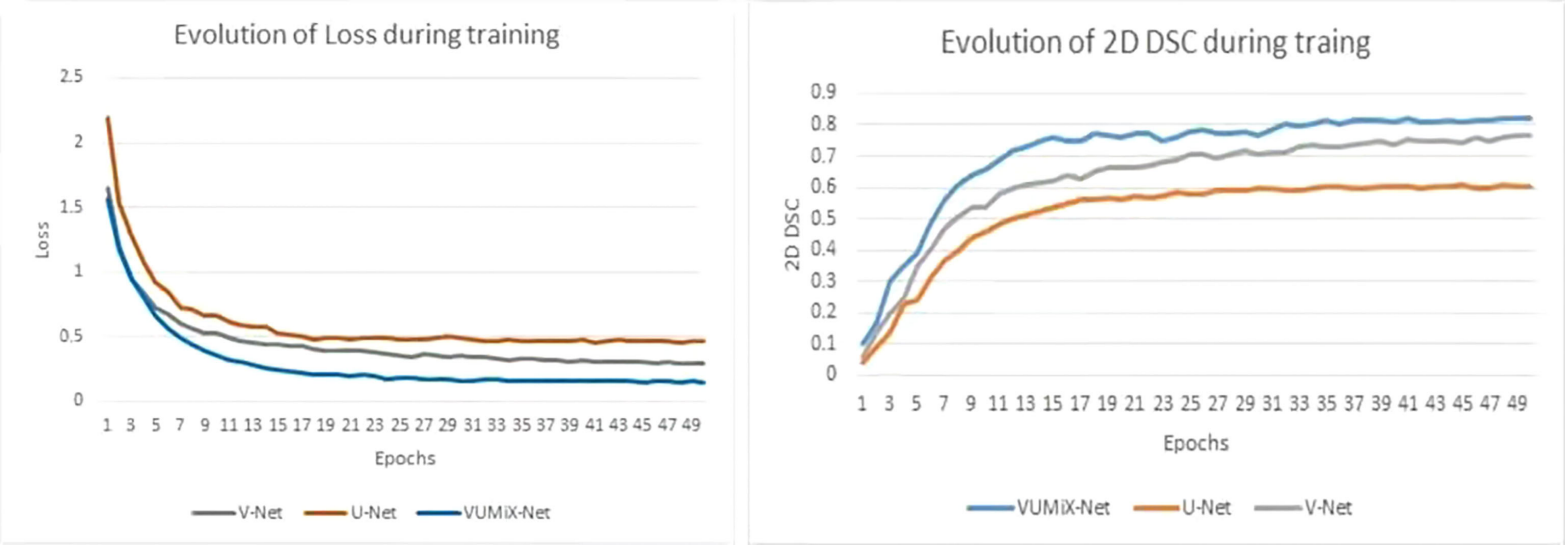
The training DSC and loss of function.

The EC localization network and segmentation network were trained in sequence. A five-fold cross-validation strategy was applied with the training–validation cohort. The model with the highest mean validation performance across the five validation data sets was selected. All the models were implemented by PyTorch and Python and trained and tested on an NVIDIA TITAN RTX with 24-GB memory. The two networks used the Adam optimizer with an initial learning rate of 0.0001 and decayed by an exponential function with gamma 0.9 for every epoch. For the localization network, we reduced the number of channels to reduce the number of parameters, the training batch size was set as 4, and the process took approximately 23 h. For the segmentation network, the training batch size was 8, and the process took approximately 15 h. To compare the performance of our proposed method, we conducted testing on the 3D segmentation network (named V-Net), the 2D segmentation network with attention enhancement (named U-Net), and VUMix-Net separately. A weighted sum of the binary cross-entropy loss and Dice loss was used as the loss function in all the segmentation networks, while binary cross-entropy loss was used in the localization network. The profiles of the training Dice similarity coefficient (DSC) and loss are shown in **Figure 3** (31).

### Quantitative Evaluation

The DSC and 95th-percentile Hausdorff distance (HD) were used to evaluate the image segmentation performance. The two values equaled 1 when the two masks were completely the same. The 95HD reflects the agreement between two contours; a higher value indicates a larger difference. The DSC was calculated at the 2D level and 3D level. Mean ± standard deviation (SD) values were calculated by averaging all the values obtained. 3D-DSC was used to compare the whole segmented volumes in three dimensions. 2D-DSC was evaluated only on the positive slices that contained manually delineated contours. The 2D-DSC was set to 0 if a positive slice was missed by the algorithm or a negative slice was incorrectly recognized as positive, which is a very rigorous evaluation method.

### Clinical Evaluation

The evaluation was conducted by two other experienced clinicians, A and B, in our center who did not participate in the GTV contouring. Both of them have more than 15 years of experience in radiotherapy and have treated more than 600 EC patients. Twenty patients were selected randomly from the clinical work. The manual reference contours were separated into the GT group, while the corresponding contours generated by the proposed model belonged to the AI group. Then, the AI results and GT results of each case were randomly labeled 1 or 2. If the AI result was labeled 1, the GT result was labeled 2 and vice versa. Two clinicians were asked to score the 1 and 2 results, slice by slice, *via* a blinded evaluation. The evaluation criteria included 4 grades: 0—rejection (the segmentation is unacceptable and needs to be redrawn); 1—major revision (the segmentation needs significant revision; 2—minor revision (the segmentation needs a few minor edits but has no significant clinical impact without correction); and 3—no revision (the segmentation is perfect and completely acceptable for treatment).

### Statistical Analysis

Categorical variables for the combined training–validation and testing cohorts were compared by using the χ2 test or Fisher exact test. One-way ANOVA analysis of variance was used to compare DSC and ASD between the different subgroups. The χ2 test was used to compare the difference in the degrees of volumetric revision between subgroups. The paired Wilcoxon rank-sum test was used to evaluate the GTV scores of two evaluators and the AI group and GT group scores of the single- evaluator clinical assessment. All statistical comparisons were performed using SPSS software (version 25.0; IBM, Inc., Armonk, NY, USA). A value of *P* < 0.05 indicated statistical significance.

## Result

### Dataset Statistical Analysis

There were 185 patients in the training–validation cohort, including 94 upper EC, 61 middle EC, and 30 lower EC patients. In the test cohort, there were 10 patients in each of the upper, middle, and lower EC groups. The patient characteristic statistical analysis results showed that no significant differences were found between the training–validation set and the test set in sex, age, and T category. The patients’ characteristics are shown in **Table 1**.

**Table 1:** Patient characteristics.

Characteristic	Entire Cohort (n = 215)	Training–ValidationCohort (n = 185)	Test Cohort (n = 30)	P-Value
Sex	Male	111	20	0.488
	Female	74	10	
Age	<60	89	14	0.833
	>60	96	16	
T category	T1-2	59	7	0.346
	T3-4	126	23	

### Segmentation Performance

**Figure 4:**
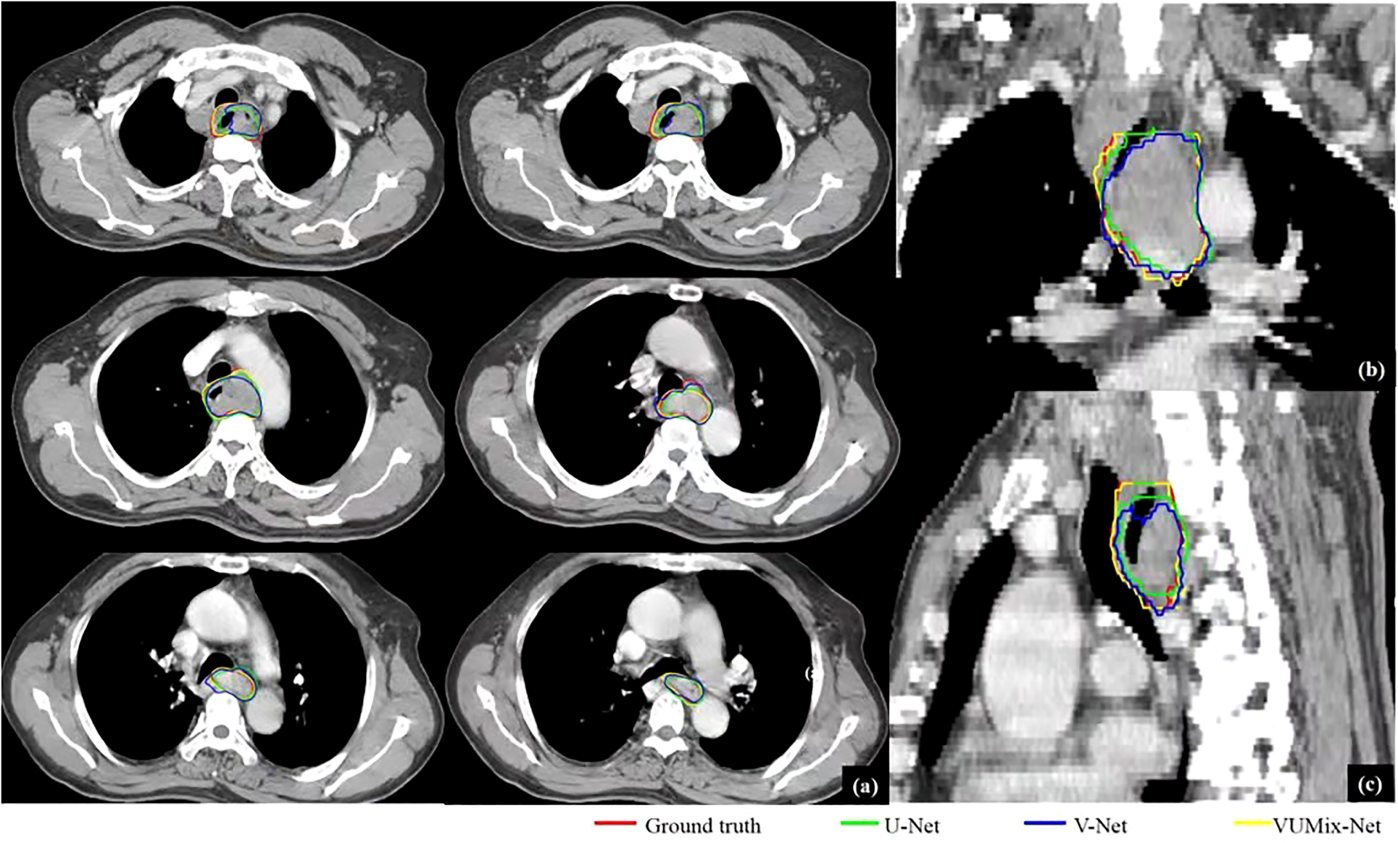
GTV delineations for a patient predicted from U-Net, V-Net, and VUMix-Net. **(A)** GTV on six transversal planes, **(B)** GTV on a coronal plane, **(C)** GTV on a sagittal plane. The CT slices were scanned from an upper esophageal cancer patient.

**Figure 5:**
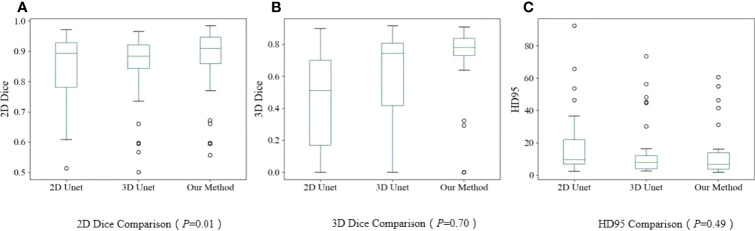
Boxplots. **(A)** 2D-DSC from U-Net, V-Net, and VUMix-Net in all patients; **(B)** 3D-DSC from U-Net, VNet, and VUMix-Net in all patients; **(C)** HD95 from U-Net, V-Net, and VUMIx-Net in all patients.

The quantitative evaluation results are summarized in **Table 2**. The mean 2D-DSCs for all GTVs from U-Net, V-Net, and VUMix-Net were 0.45 ± 0.31, 0.60 ± 0.29, and 0.68 ± 0.27, respectively. The mean 3D-DSCs were 0.84 ± 0.12, 0.84 ± 0.13, and 0.86 ± 0.12, and the mean 95HDs were 19.08 ± 21.15 mm, 15.24 ± 18.78 mm, and 13.38 ± 16.29 mm, respectively. There was a statistically significant difference in U-Net, V-Net, and VUMix-Net in terms of the 2D-DSC (*P* = 0.01). For the comparison between U-Net and VUMix-Net, the mean 2D-DSC for VUMix-Net was 0.68, which is significantly higher than that for U-Net (*P* = 0.03). VUMix-Net also tended to achieve better 3D-DSC and 95HD values than both U-Net and V-Net, although the difference was not statistically significant. **Figure 4** shows some CT slides from an EC patient with GTV delineations. **Figure 5** shows the box plots of the 2D-DSC, 3D-DSC, and 95HD values from U-Net, V-Net, and VUMix-Net across all patients.

**Table 2:** Two-dimensional Dice similarity coefficient **(**2D-DSC), three-dimensional Dice similarity coefficient **(**3D-DSC), and 95th-percentile Hausdorff distance (95HD) values of the gross tumor volume (GTV) contours in different from network architectures and esophageal locations.

	2D-DSC				3D-DSC				95HD			
	2D-DSC ± STD	P	Variance		3D-DSC ± STD	P	Variance		95HD ± STD	P	Variance	
U-Net	0.45 ± 0.31	0.01	U-V	0.07	0.84 ± 0.12	0.70	U-V	0.87	19.08 ± 21.15	0.49	U-V	0.46
V-Net	0.60 ± 0.29		V-VU	0.73	0.84 ± 0.13		V-VU	0.44	15.24 ± 18.78		V-VU	0.68
VUMiX-Net	0.68 ± 0.27		U-VU	0.00	0.86 ± 0.12		U-VU	0.52	13.38 ± 16.29		U-VU	0.25
Upper	0.72 ± 0.16	<0.001			0.90 ± 0.04	<0.001			7.95 ± 5.69	<0.001		
Middle	0.70 ± 0.20				0.92 ± 0.05				6.96 ± 4.61			
Lower	0.31 ± 0.32				0.73 ± 0.14				32.78 ± 24.23			

For all models, the mean ± STD of the 2D-DSC values for upper EC tumors (0.72 ± 0.16) were significantly higher than those for middle (0.70 ± 0.20) or lower EC tumors (0.31 ± 0.32). Additionally, the 3D-DSC values were significantly highest for middle EC tumors (0.92 ± 0.05) and lowest for lower EC tumors (0.73 ± 0.14), and there was a significant difference (*P*<0.001). Furthermore, there was a statistically significant difference in the 95HD values for upper EC, middle EC, and lower EC tumors (*P*<0.001), and middle EC tumors had the lowest values.

The 2D-DSC, 3D-DSC, and 95HD values of the upper EC and middle EC tumors were better than those of the lower EC tumors. Therefore, we specifically compared the differences among U-Net, V-Net, and VUMix-Net for upper and middle EC tumors. VUMix-Net achieved the highest 2D-DSC value (*P*<0.001) and lowest 95HD value (*P*=0.02) among these three models. Regarding the 3D-DSC value, VUMix-Net was higher than U-Net or V-Net, but the difference was not significant (**Table 3**).

**Table 3:** 2D-DSC, 3D-DSC, and 95HD values of the GTV contours in upper and middle esophageal cancer (EC) patients predicted from U-Net, V-Net, and VUMix-Net.

	2D-DSC				3D-DSC				95HD			
	2D-DSC ± STD	P	Variance		3D-DSC ± STD	P	Variance		95HD ± STD	P	Variance	
U-Net	0.58 ± 0.23	<0.001	U-V	0.01	0.90 ± 0.06	0.11	U-V	0.61	10.06 ± 6.96	0.02	U-V	0.04
V-Net	0.74 ± 0.12		V-VU	<0.001	0.91 ± 0.04		V-VU	0.06	6.37 ± 3.05		V-VU	0.03
VUMiX-Net	0.81 ± 0.06		U-VU	0.03	0.93 ± 0.03		U-VU	0.07	5.95 ± 5.75		U-VU	0.70

Contrasting the performance of the three models for upper EC, we obtained the best results with VUMix-Net Net in terms of the 2D-DSC value, 3D-DSC value, and 95HD value, but the difference was not significant. Additionally, VUMix-Net achieved the best results for middle EC, with the highest 2D-DSC value (*P*<0.001) and lowest 95HD value (*P*=0.04). Additionally, VUMix-Net obtained the highest 3D-DSC value, but the difference was not significant (*P*=0.06). In lower EC, even though VUMix-Net yielded the best results, there was no significant difference among the three models. The results are summarized in **Table 4**.

**Table 4:** 2D-DSC, 3D-DSC, and 95HD values of the GTV contours in upper EC, middle EC, and lower EC patients predicted from U-Net, V-Net, and VUMix-Net.

		2D-DSC		3D-DSC		95HD	
		2D-DSC ± STD	P	3D-DSC ± STD	P	95HD ± STD	P
Upper	U-Net	0.64 ± 0.21	0.09	0.91 ± 0.5	0.34	10.29 ± 8.33	0.29
	V-Net	0.73 ± 0.15		0.89 ± 0.03		6.73 ± 3.46	
	VUMiX-Net	0.80 ± 0.05		0.91 ± 0.03		6.84 ± 3.27	
Middle	U-Net	0.53 ± 0.25	<0.001	0.89 ± 0.07	0.06	9.82 ± 5.73	0.04
	V-Net	0.75 ± 0.09		0.92 ± 0.04		6.00 ± 2.73	
	VUMiX-Net	0.83 ± 0.06		0.94 ± 0.03		5.07 ± 3.75	
Lower	U-Net	0.19 ± 0.28	0.25	0.74 ± 0.14	0.84	37.11 ± 28.33	0.73
	V-Net	0.31 ± 0.33		0.71 ± 0.15		33.00 ± 24.33	
	VUMiX-Net	0.43 ± 0.35		0.74 ± 0.14		28.22 ± 21.47	

### Clinical Evaluation

**Figure 6:**
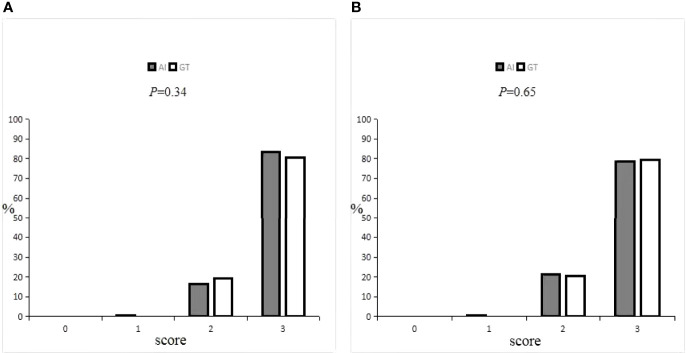
Distribution of GTV scores by **(A)** and **(B)** oncologist.

Details on the oncologists’ subjective evaluation results are given in **Tables 5**, **6**, and the score distribution is shown in **Figure 6**. The predicted GTV contours of the 440 slices from 20 patients were subjectively evaluated by the two experienced oncologists. If the score was ≥2 points, the slices could be accepted for practical, clinical applications and only needed to be slightly modified, if at all. Only 0.3% of the slices predicted by the AI system were evaluated as ‘‘major revision” among all 880 slices, obtained from 1 patient in the A oncologist group and 2 patients in the B oncologist group. In the A oncologist group, 1 and 0 slices were evaluated as ‘‘major revision” for the AI and GT groups. Additionally, the score above 2 was 83.18% and 80.68%. In the B oncologist group, 2 and 0 slices were evaluated as ‘‘major revision” for the AI and GT groups. In addition, the score above 2 was 78.41% and 79.55%. In both the A oncologist group and the B oncologist group, the grading results for the AI group were not significantly different from those for the GT group (*P*=0.34; *P*=0.65). The paired Wilcoxon rank-sum test was used to evaluate the clinical evaluations of the two oncologists, and the results showed that there was no significant difference.

**Table 5:** The oncologists’ evaluation results.

Score	A		B	
	AI	GT	AI	GT
0	0 (0.00%)	0 (0.00%)	0 (0%)	0 (0.00%)
1	1 (0.23%)	0 (0.00%)	2 (0.45%)	0 (0.00%)
2	73 (16.59%)	85 (19.32%)	93 (21.14%)	90 (20.45%)
3	366 (83.18%)	355 (80.68%)	345 (78.41%)	350 (79.55%)
P-value	0.34		0.65	

**Table 6:** Mean clinical score of GTV by two oncologists.

Score	AI		GT	
	A	B	A	B
0 1 2 3 P-value	0 (0.00%)	0 (0%)	0 (0.00%)	0 (0.00%)
	1 (0.23%)	2 (0.45%)	0 (0.00%)	0 (0.00%)
	73 (16.59%)	93 (21.14%)	85 (19.32%)	90 (20.45%)
	366 (83.18%)	345 (78.41%)	355 (80.68%)	350 (79.55%)
	0.07		0.67	

### Computational Complexity

The computational complexity of the three methods is shown in **Table 7**. The average time required to predict the segmentation results for each study was 5, 14, and 11 s, separately, using the GPU (NVIDIA TITAN RTX with 24-GB memory).

**Table 7:** Comparison of computational complexity.

Model name	Number of parameters	Size on Disk	Inference time
U-Net	7.8 M	89 MB	5 s
V-Net	67.1 M	542 MB	14 s
VUMiX-Net	13.1 M	102 MB	11 s

## Discussion

The accuracy of tumor target contouring, an important step in radiotherapy for EC, is undoubtedly closely related to the tumor control rate and the radiation dose to the surrounding normal tissues and organs. Although a global consensus has been reached on delineating the clinical target volume (CTV) in EC radiotherapy (32), we only investigated the delineation of the GTV in this study. Many studies (2, 33) have found that there is a certain degree of variability across different imaging methods following the same guidelines, in different centers, between different delineators and the delineator itself. AI has been shown to be an effective method for improving the accuracy of the delineation and reducing the variability between delineators (34). Previous studies have suggested that AI could improve the consistency and save time in the delineation of the GTV, CTV, and organs at risk in nasopharyngeal cancer (20), rectal cancer (23), breast cancer (24), and cervical cancer (26). However, no study has been conducted on automatic esophageal GTV contouring with CNN-based methods.

In our clinical experiment, we collected and standardized the CT images from 215 patients in our department and proposed a new end-to-end deep learning network named VUMix-Net to automatically segment the GTV in EC. The performance of VUMix-Net in contouring the GTV in EC was comprehensively evaluated with a comparison to the performance of U-Net and V-Net. VUMix-Net achieved a higher 2D-DSC value (p = 0.01) and tended to achieve better 3D-DSC and 95HD values than both U-Net and V-Net. The delineation results predicted from VUMix-Net were significantly more acceptable for use in radiation therapy planning than those from U-Net and V-Net.

Cervical and upper thoracic EC are difficult to operate due to the similarities in their anatomical sites (35). Therefore, radiotherapy plays a very important role in the treatment of cervical and upper thoracic EC and is more widely used than in middle or lower thoracic EC. In this study, cervical and upper thoracic EC were referred to as “upper” EC. Comparing these three segments of EC, the results showed that there was a statistically significant difference among them in terms of the 2D-DSC, 3D-DSC, and 95HD values (p<0.001); specifically, the values for upper and middle EC were better. The reasons may be as follows: first, fewer patients in the training cohort had lower EC than upper or middle EC; second, the position of the lower esophagus is easily affected by the surrounding organs and has great variability, especially at the esophagogastric junction, while the position of the middle and upper esophagus is relatively fixed.

Next, we only compared GTV contouring in upper and middle EC and further found that VUMix-net was superior to U-Net and V-Net. In addition, we continued to explore the merits and demerits of the three models in upper, middle, and lower EC, and found that VUMix-Net obtained the best results for both upper and lower EC, although the differences were not significant. In middle EC, VUMix-Net achieved the highest 2D-DSC value and lowest 95HD compared to U-Net and V-Net, and the differences were statistically significant. Therefore, this new model, VUMix-Net, had the best applicability in middle EC, followed by upper EC and finally lower EC.

It was difficult to fully reflect the segmentation quality using only single-target segmentation performance indicators such as the DSC, which is easily affected by the organ size. In the same way that delineation deviations occur at the edges of organs, the DSCs of small organs are usually lower than those of large organs (36). Considering the clinical practicability of the new model, two oncologists evaluated the results of the AI group and GT group by a blinded method and the clinical acceptability of AI delineation. The results showed that oncologists A and judged that 99.8% and 99.5%, respectively, of GTVs outlined by AI can be accepted clinically. There was no significant difference in the scores for the AI and GT groups, suggesting that the level of AI delineation was close to that of manual delineation. This result suggests that in terms of clinical work, the new model could reach a similar level of applicability to manual delineation and should be actively encouraged to use in daily clinical work.

However, a few studies (37, 38) have explored the role of deep learning in the auto-segmentation of the CTV and GTV in EC based on CT images. There are three unique novel contributions in our work. First, we presented the VUMix-Net architecture for segmenting the GTV in the planning of the neoadjuvant or radical radiation therapy of EC, instead of adjuvant radiation therapy (37). According to the guidelines, the use of neoadjuvant radiotherapy or radical radiotherapy is more widespread. Second, we compared manual delineation with AI delineation to verify the clinical application value of AI, while other studies (37, 38) have not. Third, we compared the three segments of EC and concluded that the application effect of AI was best in the middle segment, which also provides convenience for its clinical application.

There were some limitations of this study. First, it was based on single-center data, and the AI model could not fully meet the outlining principles and habits of other centers. In the future, multiple centers can jointly determine a consensus to outline standardization, obtain larger datasets and more data sources, improve the generalizability of the model, and better standardize treatments across centers. Second, the oncologist needs to combine the results of CT, gastroscopy, esophagography, and even PET-CT to outline the upper and lower bounds of the GTV, while the AI only needs to learn the delineation based on CT images, resulting in imprecise AI delineation at the upper and lower bounds. Therefore, the clinical evaluation in this study only evaluated the levels outlined by AI and GT without considering the problem of the upper and lower bounds. Subsequent research should enable the AI to learn with multimodal imaging data and improve the delineation of EC targets.

In conclusion, compared with U-Net and V-Net, the new model (VUMix-Net) showed certain advantages in the delineation of the GTVs of EC. Additionally, it can generate the GTVs of EC that meet clinical requirements and have the same quality as human-generated contours. The application effect was the best in middle-segment EC, followed by upper-segment EC, and finally lower segment EC.

## Data Availability Statement

The raw data supporting the conclusions of this article will be made available by the authors, without undue reservation.

## Ethics Statement

The study was approved by the ethics committee of Anyang Tumor Hospital, The Fourth Affiliated Hospital of Henan University of Science and Technology. The patients provided written informed consent to participate in this study.

## Author Contributions

HG and LJ designed projection and maked modifications to the paper and approved the final paper to be published. LJ, QC, AS, SW, XW, RR, and CW collected and processed datas. LJ wrote paper. AZ, NW, PS, NW, XC and YZ revised paper. All authors contributed to the article and approved the submitted version.

## Conflict of Interest

Author QC, AS, and SW were employed by MedMind Technology Co, Ltd.

The remaining authors declare that the research was conducted in the absence of any commercial or financial relationships that could be construed as a potential conflict of interest.

## Publisher’s Note

All claims expressed in this article are solely those of the authors and do not necessarily represent those of their affiliated organizations, or those of the publisher, the editors and the reviewers. Any product that may be evaluated in this article, or claim that may be made by its manufacturer, is not guaranteed or endorsed by the publisher.
